# Prognostic Value of Neutrophil Percentage–Albumin Ratio in Patients with Advanced Melanoma Treated with Immune Checkpoint Inhibitors

**DOI:** 10.3390/curroncol33060302

**Published:** 2026-05-22

**Authors:** Emre Eken, Emel Ayvaz Güneyin, Elif Büyükkurt, Faruk Yıldız, Mehmet Bilici, Canan Dinar Ayman

**Affiliations:** 1 Department of Medical Oncology, Faculty of Medicine, Atatürk University, 25240 Erzurum, Türkiye; emel.ayvaz@atauni.edu.tr (E.A.G.); e.buyukkurt@atauni.edu.tr (E.B.); farukyildiz@grv.atauni.edu.tr (F.Y.); mbilici@atauni.edu.tr (M.B.); 2 Department of Internal Medicine, Faculty of Medicine, Atatürk University, 25240 Erzurum, Türkiye; dinarcanan@gmail.com

**Keywords:** melanoma, neutrophil percentage–albumin ratio, immunotherapy

## Abstract

Background: Although immune checkpoint inhibitors (ICIs) have improved survival in advanced melanoma, predicting individual responses remains challenging; thus, practical and inexpensive biomarkers are needed. In this study, we investigated the prognostic value of the neutrophil percentage–albumin ratio (NPAR) in patients with advanced melanoma receiving ICI therapy. Methods: Fifty patients treated in our clinic were included, with a mean age of 53.3 years and 66% being male. Visceral metastases were present in 76% of the cohort. Through conducting Receiver Operating Characteristic (ROC) analysis, we determined an NPAR cut-off value of 1.81, with patients categorized into low (<1.81, *n* = 27)- and high (≥1.81, *n* = 23)-NPAR groups. The progression-free survival (PFS) and overall survival (OS) were evaluated using Kaplan–Meier and Cox regression analyses. Results: High NPAR (≥1.81) significantly shortened both PFS and OS. In the univariate analysis, high NPAR emerged as a strong risk factor for PFS (HR: 2.68, *p* = 0.002) and OS (HR: 3.70, *p* < 0.001), while multivariate analysis confirmed NPAR as an independent negative prognostic factor for PFS (HR: 2.45, *p* = 0.006) and OS (HR: 2.82, *p* = 0.003), regardless of clinical variables. Additionally, visceral metastasis was an independent negative predictor of survival. Conclusions: Pre-treatment NPAR levels may be an independent and potential predictor of survival in advanced melanoma patients receiving ICIs. This easily calculable ratio could provide a practical guide for risk stratification.

## Simple Summary

In this study, we predicted that a simple value, the Neutrophil Percentage-to-Albumin Ratio (NPAR), could predict the prognosis of patients with advanced-stage melanoma receiving immunotherapy. Although immunotherapy is very effective in treating this malignancy, it is difficult to know in advance which patients will respond favorably to the treatment; therefore, there is a need for inexpensive and reliable methods that can be easily applied with routine blood tests. In this study, an NPAR value of 1.81 and above was determined as signifying high risk. It was observed that in patients with high NPAR values, both survival and progression-free survival (PFS) times were significantly shorter compared to those with low values, and the risk of death was approximately 3 times higher in patients with high NPAR levels. Consequently, NPAR appears to be a simple, cost-effective, non-invasive, and practical method that could assist doctors in monitoring a patient’s condition and making treatment decisions.

## 1. Introduction

Melanoma is an aggressive neoplasm arising from the uncontrolled proliferation of melanocytes that synthesize melanin pigment, which occurs primarily in the skin where melanocytes are densely located, as well as in the eye, ear, gastrointestinal system, leptomeninges, and mucosal surfaces (oral, genital, sinus) [1,2,3]. Although it constitutes approximately 1% of skin cancers, it is responsible for most deaths in this group owing to its metastatic potential and aggressive course [3,4,5]. Exposure to ultraviolet (UV) radiation (sunlight or artificial tanning) is involved in its etiology, which explains its increased incidence in fair-skinned individuals [1,6].

Melanoma shows a marked tendency for lymphatic spread, with regional lymph nodes frequently representing the first site of metastasis. As the disease progresses, distant organ involvement becomes evident, most commonly affecting the lungs, brain, liver, and bones [7,8,9]. While surgical resection offers a potentially curative treatment option for early-stage disease, in the metastatic stage, chemo-, radio-, immuno-, and targeted therapies are used either together or sequentially [10]. Historically, cytotoxic chemotherapy regimens such as dacarbazine, temozolomide, or the CVD (cisplatin, vinblastine, and dacarbazine) combination have been used; however, owing to the chemoresistant nature of melanoma, these treatments have provided limited benefit for survival and have been associated with significant toxicities [11,12,13].

The recognition that melanoma is a tumor characterized by prominent lymphocyte infiltration and is capable of strongly stimulating the immune system has fundamentally changed the approach to treating this cancer [2,10]. Currently, ICIs targeting the programmed cell death protein-1 (PD-1) pathway are the basis of systemic treatment for metastatic melanoma. Various anti-PD-1 antibodies, particularly nivolumab and pembrolizumab, are used alone or in combination with anti-CTLA-4 and anti-LAG-3 agents, maintaining their place among standard treatment options in current guidelines [2,4,14]. In contrast, in somatic BRAF mutations (most commonly V600E), which are observed in approximately half of melanomas associated with sun exposure, combinations of BRAF and MEK inhibitors can be used [3,6]. However, it should also be emphasized that the benefit of ICIs has been demonstrated in both mutation-positive and wild-type patients, independent of BRAF mutation status [1,15,16,17].

In Türkiye, the clinical application of systemic therapies is primarily guided by the Social Security Institution (SGK) reimbursement regulations. While current guidelines recommend ICIs across various lines, national policies in place during the study period often required specific prior treatment failures or clinical criteria for their reimbursement. Consequently, our cohort reflects a real-world scenario in which treatment sequencing, including the use of ICIs in second- or third-line settings, was influenced by these regulatory frameworks and drug access.

Systemic inflammation plays a critical role in tumor progression, metastasis, and response to treatment. In this context, hematological parameters reflecting the inflammatory status of patients, such as the neutrophil–lymphocyte ratio (NLR) and systemic immune-inflammation index (SII), have been shown to be independent prognostic markers in many solid tumors, including metastatic melanoma. In addition, lactate dehydrogenase (LDH), which reflects tumor burden and aggressive disease biology, is widely used in clinical practice and included in staging systems. A high baseline inflammatory burden has been associated with worse survival outcomes in various meta-analyses [18,19,20,21,22], particularly in patients treated with ICIs. However, these biomarkers have several limitations, including a lack of specificity, inter-patient variability, and inconsistent predictive performance across different clinical settings.

In recent years, beyond these conventional biomarkers, researchers have not only examined inflammatory parameters but have also increasingly focused on combined biomarkers that reflect patients’ nutritional status and overall physiological reserve. At this point, two parameters are particularly noteworthy: neutrophils, which have been associated with tumor progression and systemic inflammatory responses, and serum albumin, which reflects both systemic inflammation and nutritional status of the patient. Integrating these two markers may provide a more comprehensive assessment of the inflammatory and nutritional status of cancer patients [23,24]. A product of this approach, the Neutrophil Percentage/Albumin Ratio (NPAR) combines inflammatory burden and systemic reserve into a single score and may emerge as an easy to use and promising parameter in clinical practice [25,26,27].

Although the prognostic significance of NPAR has been investigated in various solid tumors, its clinical importance has not yet been sufficiently demonstrated in metastatic melanoma, particularly in patients receiving ICIs. However, in the current era of immunotherapy, the need for practical, cost-effective, and reproducible biomarkers is increasingly evident; such parameters both strengthen risk stratification and allow for more rational implementation of treatment strategies. Based on these considerations, we aimed to investigate the prognostic value of baseline Neutrophil Percentage/Albumin Ratio (NPAR) levels on OS and PFS in patients with metastatic melanoma receiving ICI therapy.

## 2. Materials and Methods

### 2.1. Study Design and Population

We analyzed a cohort of patients diagnosed with metastatic melanoma between 1 January 2012 and 1 January 2026 who had previously received immunotherapy. This single-center study was conducted at Atatürk University, with approval from the Atatürk University Faculty of Medicine Non-Interventional Clinical Research Ethics Committee (Decision No: B.30.2.ATA.0.01.00/102, Date: 26 February 2026), and the study was conducted in accordance with the principles of the Declaration of Helsinki. All participants were aged ≥18 years. Owing to the retrospective nature of the study and the use of de-identified data, the requirement for informed consent was waived by the institutional ethics committee.

### 2.2. Inclusion and Exclusion Criteria

Patients aged ≥ 18 years with histopathologically confirmed metastatic melanoma who had received at least one course of immunotherapy (nivolumab, ipilimumab, or combination therapy) were included. Missing data were handled by listwise deletion; specifically, patients with missing baseline laboratory parameters or incomplete clinical follow-up data were excluded from the final analysis to ensure the integrity of statistical models.

### 2.3. Data Collection and Definitions

Demographic characteristics (age, sex), clinical data (BRAF mutation status, metastatic sites, ECOG performance status, line of treatment, administered immunotherapy regimen), and laboratory parameters were obtained from patient records. Visceral metastasis was defined as the involvement of internal organs, such as the lungs, liver, or brain, whereas non-visceral metastasis included involvement of the skin, subcutaneous tissues, or distant lymph nodes. Serum neutrophil percentage and albumin levels were recorded from blood samples obtained within seven days before treatment initiation. The Neutrophil Percentage/Albumin Ratio (NPAR) was calculated by dividing the neutrophil percentage by the serum albumin level. For LDH, values ≥ 250 U/L were considered high.

### 2.4. Study Endpoints

OS was defined as the time from the initiation of immunotherapy to the date of death, and PFS from the initiation to the date of radiological progression or death. Patients without observed progression or death were censored at their last follow-up.

### 2.5. Statistical Analysis

All statistical analyses were performed using SPSS v27.0 (IBM Corp., Armonk, NY, USA) and R v4.5.2 (R Foundation for Statistical Computing, Vienna, Austria).

Categorical variables were expressed as numbers and percentages (%) and continuous variables as mean ± standard deviation (SD) or median (minimum–maximum) according to distribution characteristics.

Pearson’s Chi-square or Fisher’s exact test were used to compare baseline characteristics between NPAR groups. ECOG performance status was considered a categorical variable; when evaluating its effect on survival, ECOG 0 was considered the reference group.

ROC curve analysis was used to determine the optimal cutoff value for NPAR, and the most appropriate threshold was calculated based on the Youden index. The sensitivity and specificity values for this threshold were reported.

Survival differences between groups were analyzed using the Kaplan–Meier method and statistically compared using the log-rank test. Prognostic factors that could affect PFS and OS were first evaluated with univariate and then multivariate Cox proportional hazards regression models, with the proportional hazards assumption verified using Schoenfeld residuals via the “survival” package in R and log-minus-log survival curves, and no significant violations were detected. To avoid overfitting and ensure the parsimony of the statistical models, variables with *p*-values < 0.05 in the univariate analysis were subsequently included in the multivariate Cox regression models. The results are presented as hazard ratios (HRs) with 95% confidence intervals (CIs).

The discriminative ability of the multivariate models was measured using Harrell’s C-index, and internal validation of the model was performed using the bootstrap method with 1000 repetitions. The optimism-corrected C-index values obtained from the bootstrap analyses are also reported among the findings.

Finally, the performance of NPAR in predicting 12-month PFS and OS was evaluated using time-dependent ROC analysis, and the area under the curve (AUC) values were calculated.

The “survival” and “timeROC” packages in R software were used for survival analyses and graph generation.

In all analyses, a two-sided *p*-value < 0.05 was considered statistically significant.

### 2.6. Visualization and Description of Figures

**Figure 1:**
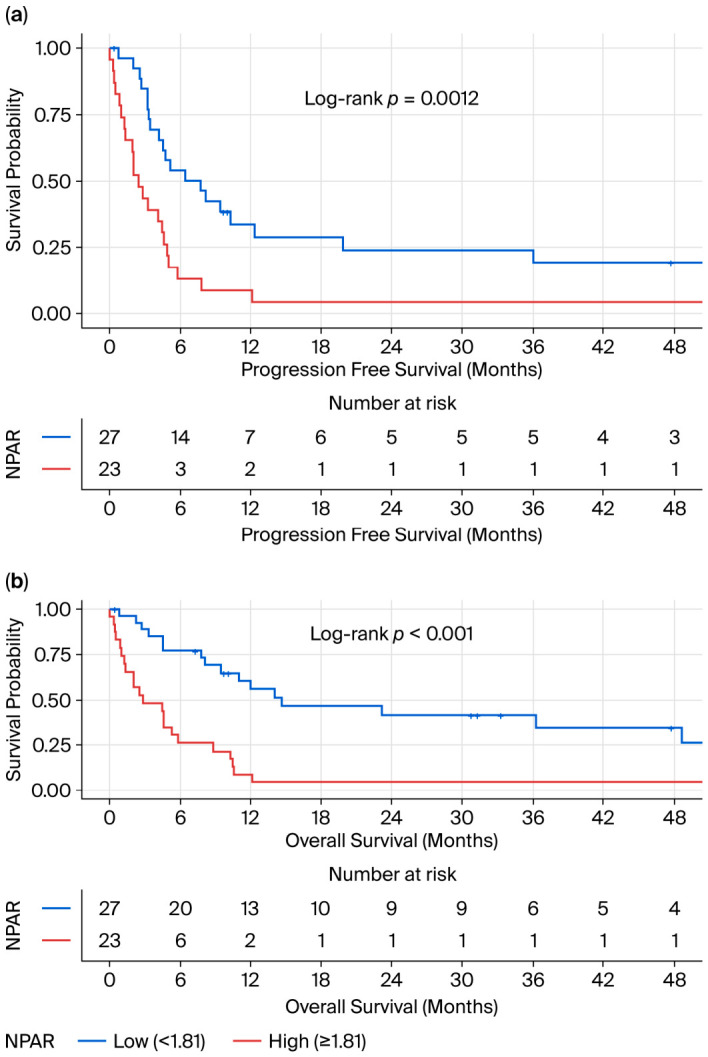
(**a**) Kaplan–Meier curves for progression-free survival (PFS) stratified by NPAR levels (cut-off: 1.81) in patients with metastatic melanoma (log-rank *p* = 0.0012). (**b**) Kaplan–Meier curves for overall survival (OS) stratified by NPAR levels (cut-off: 1.81) in patients with metastatic melanoma (log-rank *p* < 0.001).

**Figure 2:**
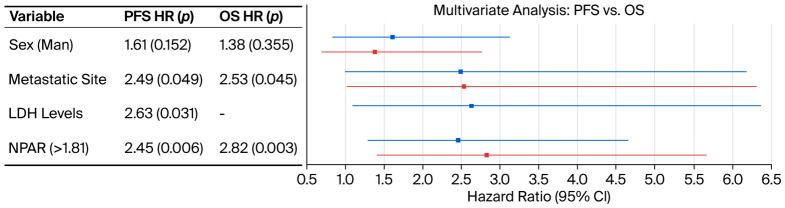
Forest plot of multivariate Cox regression analysis for progression-free (PFS) and overall survival (OS). NPAR (>1.81) emerged as an independent prognostic factor for both PFS (HR: 2.45, *p* = 0.006) and OS (HR: 2.82, *p* = 0.003). Blue lines represent progression-free survival (PFS), and red lines represent overall survival (OS).

**Figure 3:**
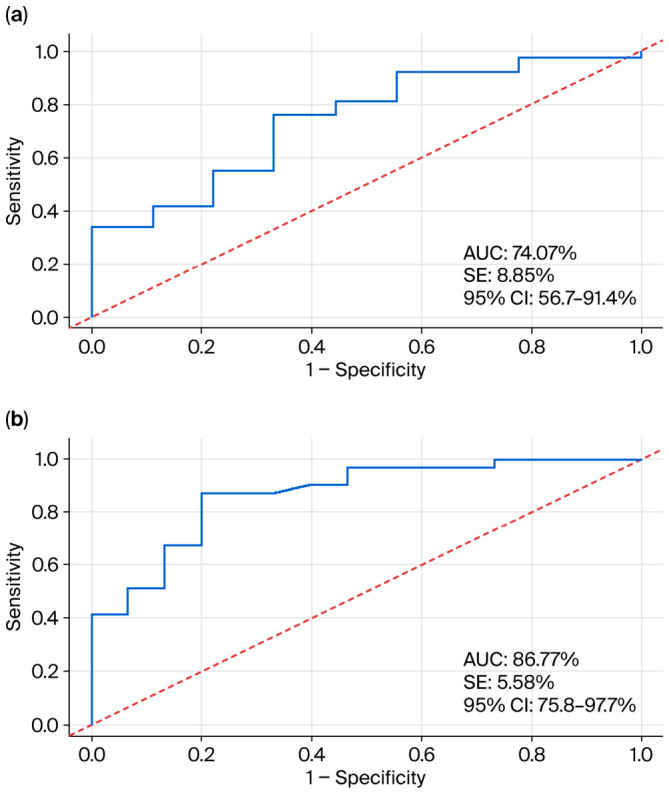
(**a**) Time-dependent ROC curve analysis of NPAR for predicting 12-month progression-free survival. The area under the curve (AUC) was 74.07% (95% CI: 56.7–91.4), indicating moderate-to-good predictive accuracy. (**b**) Time-dependent ROC curve analysis of NPAR for predicting 12-month overall survival. The area under the curve (AUC) was 86.77% (95% CI: 75.8–97.7), demonstrating a high predictive performance for long-term survival outcomes. The solid blue line represents the ROC curve for NPAR, and the dashed red line represents the reference line.

The prognostic and predictive performance of NPAR was visualized through multiple graphical representations. The PFS and OS differences between the NPAR groups are presented using Kaplan–Meier curves (Figure 1). The independent prognostic impact of clinical variables and NPAR is illustrated in the multivariate Cox regression forest plot in Figure 2. Furthermore, the time-dependent predictive accuracy of the NPAR for survival was evaluated and displayed using time-dependent Receiver Operating Characteristic (ROC) curves in Figure 3a (for PFS) and Figure 3b (for OS). Statistical significance for all survival comparisons was assessed using the log-rank test.

## 3. Results

### 3.1. Baseline Patient Characteristics

The demographic and clinical characteristics of the 50 patients included in the study are shown in Table 1. Briefly, the mean patient age was 53.26 ± 14.93 years, and 66% were male. According to BRAF mutation analysis, 18.4% of the patients had a V600E mutation, while 81.6% had wild-type tumors. When metastatic sites were examined, visceral organ metastasis was detected in 76% of patients and non-visceral (bone and lymph node) metastasis in 24%.

### 3.2. Performance Status and Treatment Profiles

When ECOG performance status was evaluated, no statistically significant difference was found between the NPAR groups (*p* = 0.151) (Table 1). Regarding treatment, immunotherapy regimens included nivolumab, ipilimumab, and nivolumab + ipilimumab combination therapy, administered across different treatment lines, as detailed in Table 1.

### 3.3. Survival Outcomes for the Entire Cohort

At the end of the follow-up period, disease progression developed in 86% (*n* = 43) of the patients, and 39 had died. The median PFS for the entire cohort was 4.5 months, and the median OS was 8.1 months.

### 3.4. Prognostic Value of NPAR on Survival

The optimal cut-off value for NPAR was determined to be 1.81 by ROC analysis (sensitivity: 59%; specificity: 100%), according to which we divided patients into low (<1.81) and high (≥1.81) NPAR groups. This optimal cut-off value was found to be identical for both OS and PFS. When survival durations were examined according to NPAR groups, the median PFS was 6.5 months (95% CI: 4.24–19.91) in the low-NPAR group, while it was 2.5 months (95% CI: 1.38–4.96) in the high-NPAR group (log-rank *p* = 0.001) (Figure 1). Similarly, the median OS was 14.6 months (95% CI: 9.53–NA) in the low-NPAR group, whereas it was 2.8 months (95% CI: 1.38–8.84) in the high-NPAR group (log-rank *p* < 0.001) (Figure 2).

All patients in the high-NPAR group experienced disease progression, and there were no surviving patients at the end of follow-up. The survival differences between the NPAR groups were statistically significant (Table 2).

**Table 2:** Univariate and multivariate Cox proportional hazards analyses of predictive factors for progression-free survival (PFS) and overall survival (OS).

		PFS				OS			
		Univariate		Multivariate		Univariate		Multivariate	
		HR %95 CI	*p* Value	HR %95 CI	*p* Value	HR %95 CI	*p* Value	HR %95 CI	*p* Value
Sex	Woman	1.97 (1.038–3.769)	**0.038**	1.61 (0.83–3.12)	0.152	2.16 (1.10–4.25)	**0.024**	1.38 (0.69–2.76)	0.355
	Man								
Age	<65	1.14 (0.572–2.282)	0.707			1.062 (0.53–2.11)	0.864		
	≥65								
Treatment Group	Nivolumab	0.73 (0.35–1.51)	0.693			0.675 (0.303–1.503)	0.564		
	Ipilimumab	0.81 (0.34–1.92)				0.913 (0.370–2.256)			
	Nivolumab + İpilimumab (Ref)	1				1			
Line of Therapy	First-line (Ref)	1				1	0.655		
	Second-line	1.67 (0.75–3.71)				1.43 (0.61–3.36)			
	Third-line	1.71 (0.64–4.60)	0.410			1.53 (0.55–4.23)			
Metastatic Site	Visceral		**<0.001**	2.49 (1.002–6.188)	**0.049**	3.55 (1.42–8.85)	**0.006**	2.53 (1.02–6.31)	**0.045**
	Non-visceral	4.51 (1.84–11.09)							
BRAF Mutation Status	V600E-Positive	0.75 (0.36–1.58)	0.459			0.98 (0.43–2.25)	0.973		
	Negative								
ECOG Status	ECOG 0 (Ref)	1	0.605			1	0.827		
	ECOG 1	0.80 (0.36–1.75)				0.81 (0.35–1.83)			
	ECOG 2	1.20 (0.60–2.42)				1.04 (0.50–2.18)			
LDH Levels	<250	2.99 (1.30–6.86)	**0.010**	2.63 (1.09–6.36)	**0.031**	2.20 (0.95–5.09)	0.064		
	≥250								
NPAR	<1.81	2.68 (1.44–5.00)	**0.002**	2.45 (1.29–4.65)	**0.006**	3.70 (1.88–7.26)	**<0.001**	2.82 (1.41–5.66)	**0.003**
	≥1.81								
Model C-index					0.739				0.742

Note: Bold values indicate statistically significant results (*p* < 0.05). Internal validation of the multivariate Cox model for PFS was performed using a bootstrapping method with 1000 repetitions. The original C-index was 0.739, and the bias-corrected C-index was 0.726, indicating high predictive accuracy. Internal validation of the multivariate Cox model for OS was performed using the bootstrapping method with 1000 repetitions. The original C-index was 0.742, and the bias-corrected C-index was 0.7293, indicating a high predictive accuracy. Abbreviations: NPAR, neutrophil percentage-to-albumin ratio; ECOG, Eastern Cooperative Oncology Group Performance Status Scale.

## 4. Discussion

Our results suggest that the neutrophil percentage–albumin ratio (NPAR), calculated before treatment, may be associated with both PFS and OS in patients with advanced malignant melanoma treated with ICIs. In multivariate analyses, patients with NPAR ≥ 1.81 had a 2.45-fold higher risk of progression and a 2.82-fold higher risk of death than those in the low-NPAR group. These findings support the potential prognostic value of NPAR in patients undergoing immunotherapy.

The association between NPAR and survival outcomes in melanoma patients receiving immunotherapy may be related to systemic inflammation and nutritional status. Elevated neutrophil levels have been associated with systemic inflammation and poor clinical outcomes in previous studies [28]. Similarly, hypoalbuminemia has been linked to impaired nutritional and systemic statuses and poorer outcomes in patients receiving immunotherapy [29]. Therefore, a high NPAR value may reflect an unfavorable systemic inflammatory and immunonutritional profile associated with poorer survival outcomes in ICI-treated patients.

Many studies support our findings regarding NPAR’s prognostic value of NPAR. In their analysis of adult-type diffuse gliomas, Zhu et al. identified NPAR as the top prognostic factor, outperforming traditional markers in survival prediction [30]. In colorectal cancer, Xie et al. showed that a high NPAR negatively affects PFS and OS, which is consistent with our results [31]. Özbilgeç et al. found that a high NPAR was significantly associated with survival, disease stage, and myometrial invasion in endometrial carcinoma [32]. In bladder cancer, Ferro et al. highlighted NPAR’s success of NPAR in predicting mortality post-neoadjuvant chemotherapy and its potential for clinical decision-making [33]. Recent studies on other advanced malignancies have underscored the prognostic significance of systemic inflammatory markers. For example, pretreatment inflammatory indices were independent survival predictors in patients with advanced renal cell carcinoma treated with combination immunotherapy [34].

Compared with existing prognostic scores for melanoma management, NPAR is a simple biomarker with potential clinical significance. Öksüz et al. showed that the GRIm score, including LDH, NLR, and albumin, predicted treatment response in melanoma patients on nivolumab [35]; however, in our study, NPAR showed comparable risk stratification performance with fewer parameters. The HALP score, evaluated by Ata et al., emphasizes the prognostic relevance of immune-nutritional status by combining hemoglobin, albumin, lymphocytes, and platelets [36]. In comparison, NPAR requires only two components, which may facilitate its clinical application. Using real-world data, Keskin and Karadurmuş showed that a higher inflammatory burden is associated with poorer survival in advanced melanoma patients [37]. Our findings suggest that NPAR may partially reflect inflammatory and nutritional status. In contrast, in studies on ICI response in a Japanese patient population, Horisaki et al. reported that the CONUT score and modified Glasgow Prognostic Score (mGPS) were associated with treatment outcomes [38,39]. Overall, NPAR may represent a simpler alternative to more complex inflammation- and nutrition-based prognostic scores in patients with melanoma receiving immunotherapy.

Although NPAR emerged as a significant prognostic factor, the potential impact of confounding clinical factors should be considered. ECOG performance status, pre-treatment LDH levels, and therapy line influence survival outcomes in patients with metastatic melanoma receiving ICIs, with similar findings in real-world studies on prognostic factors in advanced melanoma [40]. Although multivariable models were adjusted for important predictors, heterogeneity in treatment lines, largely driven by local healthcare reimbursement policies, remains crucial. In Türkiye, immunotherapy and its sequencing are generally determined by specific regulatory criteria, potentially resulting in different disease stages compared with other international cohorts. These system-related factors, combined with individual patient characteristics, such as baseline inflammatory burden, underscore the complexity of prognostic modeling in real-world settings. A comparable baseline distribution between the pre-treatment and high-NPAR groups is expected, as NPAR is a prognostic biomarker for risk stratification, not a determinant of group allocation or disease severity at baseline.

Bootstrap analysis was performed to assess the robustness of the prognostic model, supporting the internal validity of our findings and their potential applicability to different patient groups. The concordance index (C-index) indicated good discriminatory performance of the NPAR in stratifying patients into risk groups during follow-up. In time-dependent ROC analyses, NPAR showed an AUC of 74.07% for 12-month PFS (Figure 3a) and 86.77% for 12-month OS (Figure 3b), suggesting a better discriminative performance for OS than for PFS over the 12-month period.

In daily oncology practice, NPAR may serve as a practical adjunctive tool. A higher pre-treatment NPAR (≥1.81) may help identify patients at an increased risk. In such cases, closer radiological follow-up and/or monitoring of nutritional and inflammatory status before and during ICI therapy may be considered. Although NPAR should not replace standard clinical assessments, its potential integration into routine evaluations may support individualized follow-up strategies.

Although our findings provide preliminary support for the prognostic value of NPAR, several methodological limitations must be considered. First, the single-center nature, retrospective design, and small sample size of the study limit its generalizability and require external validation with an independent cohort. The high mortality and progression rates in the high-NPAR group should be interpreted cautiously, as they may indicate a separation effect or small-sample bias. Given the small sample size and potential events-per-variable (EPV) constraints, we used a parsimonious approach that included only significant univariate predictors. While the cutoff value’s internal validity and robustness against optimistic bias were confirmed by the optimism-corrected bootstrap method with 1000 repetitions, supporting the model’s performance in different patient groups with prospective studies will enhance our findings’ reliability. Additionally, the healthcare reimbursement conditions in Türkiye allowed patients to receive immunotherapy at different treatment lines, a potential confounding factor affecting patient profiles and survival outcomes, which should be considered in the interpretation. The long inclusion period (2012–2026) spans different therapeutic eras, introducing potential time-related selection bias. The cohort included patients treated with varying ICI regimens, such as anti-PD-1 monotherapy, anti-CTLA-4 monotherapy, and combination therapy, which may have influenced survival outcomes independently of NPAR levels. Finally, potential population differences, including genetic backgrounds and regional clinical practices, may limit the direct applicability of our NPAR threshold to different ethnic or geographic cohorts. Furthermore, detailed treatment response categories (e.g., CR, PR, and SD) were not consistently available owing to the retrospective design, limiting further response-based analyses.

## 5. Conclusions

In this study, we suggest that the pre-treatment NPAR (Neutrophil Percentage–Albumin Ratio) level is an independent, low-cost, and potentially reliable biomarker associated with survival in patients with advanced melanoma receiving ICIs. The identified threshold value of ≥1.81 may provide clinicians with a preliminary reference point for distinguishing patients at high risk of progression and mortality.

Compared with complex multi-component scoring systems, the NPAR stands out because of its ease of use in routine clinical practice and strong predictive ability. In this respect, high NPAR values may be interpreted as an “early warning sign” for re-evaluating the treatment strategy and for closer patient follow-up in the future. However, given the retrospective design and the limited cohort size, these conclusions should be interpreted with caution. Therefore, NPAR may represent a modest but potentially useful prognostic tool for risk stratification in patients receiving ICIs, although further large-scale prospective studies are needed to validate its clinical utility.

## Figures and Tables

**Table 1:** Baseline demographic and clinical characteristics of the patient cohort.

Variables	Overall Cohort	NPAR < 1.81	NPAR ≥ 1.81	*p* Value
Total, *n* (%)	50	27 (54)	23 (46)	
Mean Age, Years (SD)	53.26 (14.93)	52.11 (15.2)	54.61 (14.8)	0.561 ^b^
Gender, *n* (%)				
Males	33 (66.0)	20 (74.1)	13 (56.5)	0.192 ^b^
Females	17 (34.0)	7 (25.9)	10 (43.5)	
BRAF Mutation Status, *n* (%)				
V600E-Positive	9 (18.4)	4 (15.4)	5 (21.7)	0.716 ^a^
Negative	40 (81.6)	22 (84.6)	18 (78.3)	
Metastatic Site, *n* (%)				
Visceral	38 (76.0)	18 (66.7)	20 (87.0)	0.094 ^b^
Non-visceral	12 (24.0)	9 (33.3)	3 (13.0)	
Treatment Group, *n* (%)				
Nivolumab	26 (52.0)	16 (59.3)	10 (43.5)	0.538 ^b^
Ipilimumab	11 (22.0)	5 (18.5)	6 (26.1)	
Nivolumab + Ipilimumab	13 (26.0)	6 (22.2)	7 (30.4)	
Line of Therapy, *n* (%)				
First-line	14 (28.0)	9 (33.3)	5 (21.7)	0.407 ^a^
Second-line	27 (54.0)	15 (55.6)	12 (52.2)	
Third-line	9 (18.0)	3 (11.1)	6 (26.1)	
ECOG Status				
ECOG 0	18 (36.0)	12 (44.4)	6 (26.1)	0.151 ^b^
ECOG 1	15 (30.0)	9 (33.3)	6 (26.1)	
ECOG 2	17 (34.0)	6 (22.2)	11 (47.8)	
Progression Status, *n* (%)				
Progressive	43 (86.0)	20 (74.1)	23 (100.0)	**0.011 ^a^**
Non-progressive	7 (14.0)	7 (25.9)	0 (0.0)	
Survival Status, *n* (%)				
Alive	11 (22.0)	11 (40.7)	0 (0.0)	**<0.001 ^a^**
Death	39 (78.0)	16 (59.3)	23 (100.0)	

Note: Bold values indicate statistically significant results (*p* < 0.05). Abbreviations: NPAR, Neutrophil Percentage-to-Albumin Ratio; ECOG, Eastern Cooperative Oncology Group Scale; ^a^ Fisher’s Exact Test; ^b^ Pearson’s chi-square test. BRAF: B-Raf proto-oncogene, serine/threonine kinase.

## Data Availability

The data presented in this study are available on request from the corresponding author due to ethical and privacy restrictions related to patient data.
